# Supplementary figures and images for: Doxorubicin induces prolonged DNA damage signal in cells overexpressing DEK isoform-2
Source: PLoS One. 2022 Oct 3;17(10):e0275476. doi: 10.1371/journal.pone.0275476 (PMC9529144; doi:10.1371/journal.pone.0275476)

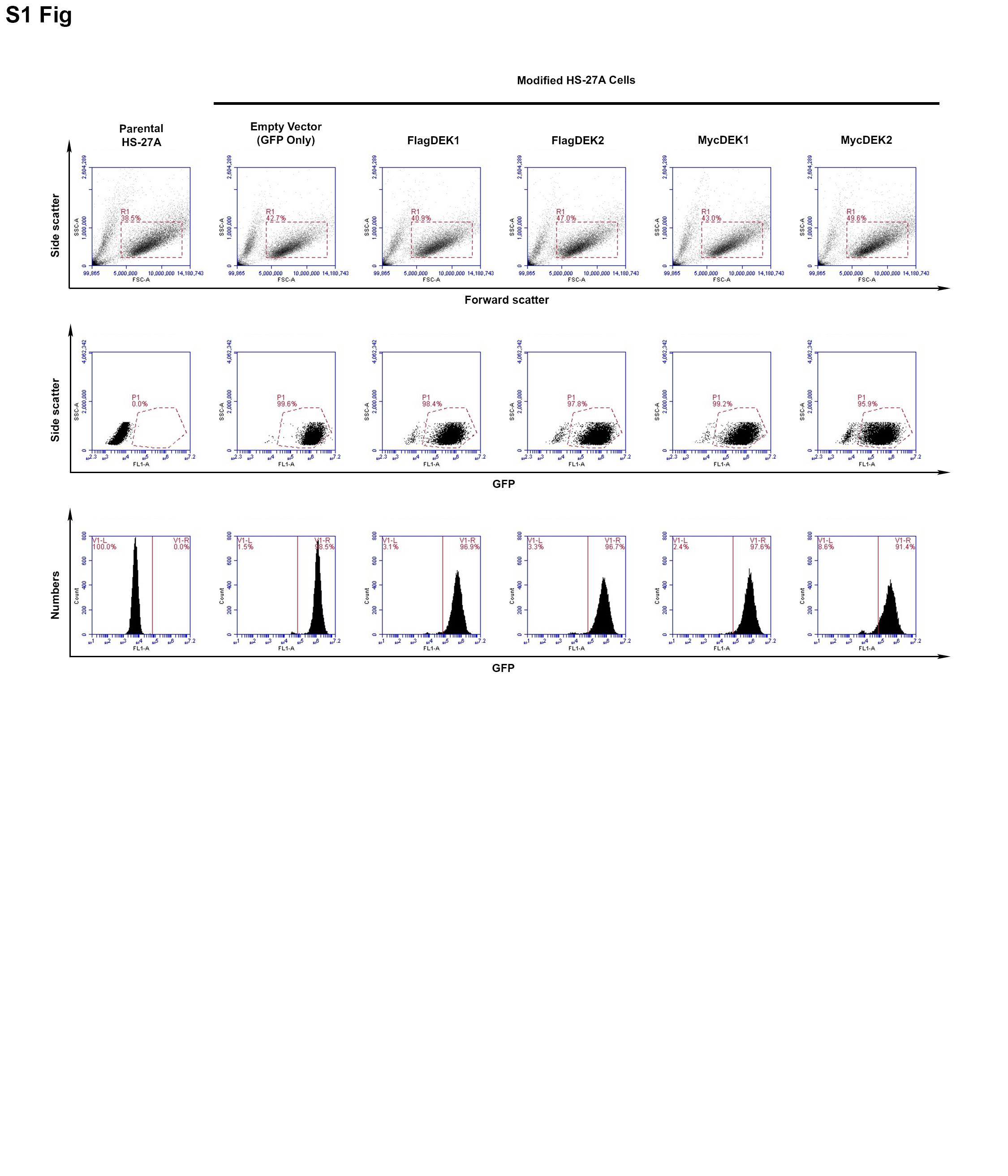

Supplement: S1 Fig — (TIF) [file pone.0275476.s001.tif]

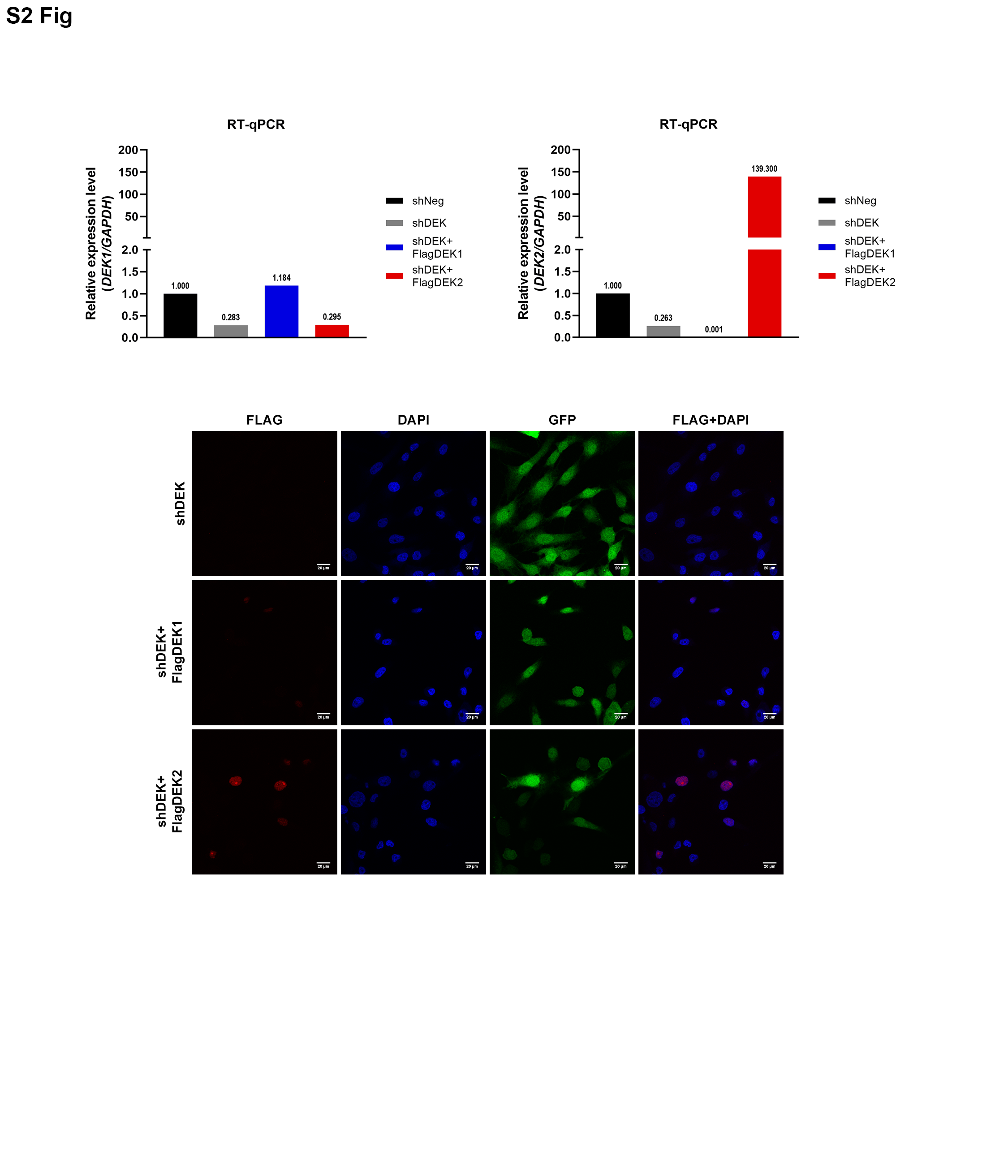

Supplement: S2 Fig — (TIF) [file pone.0275476.s002.tif]

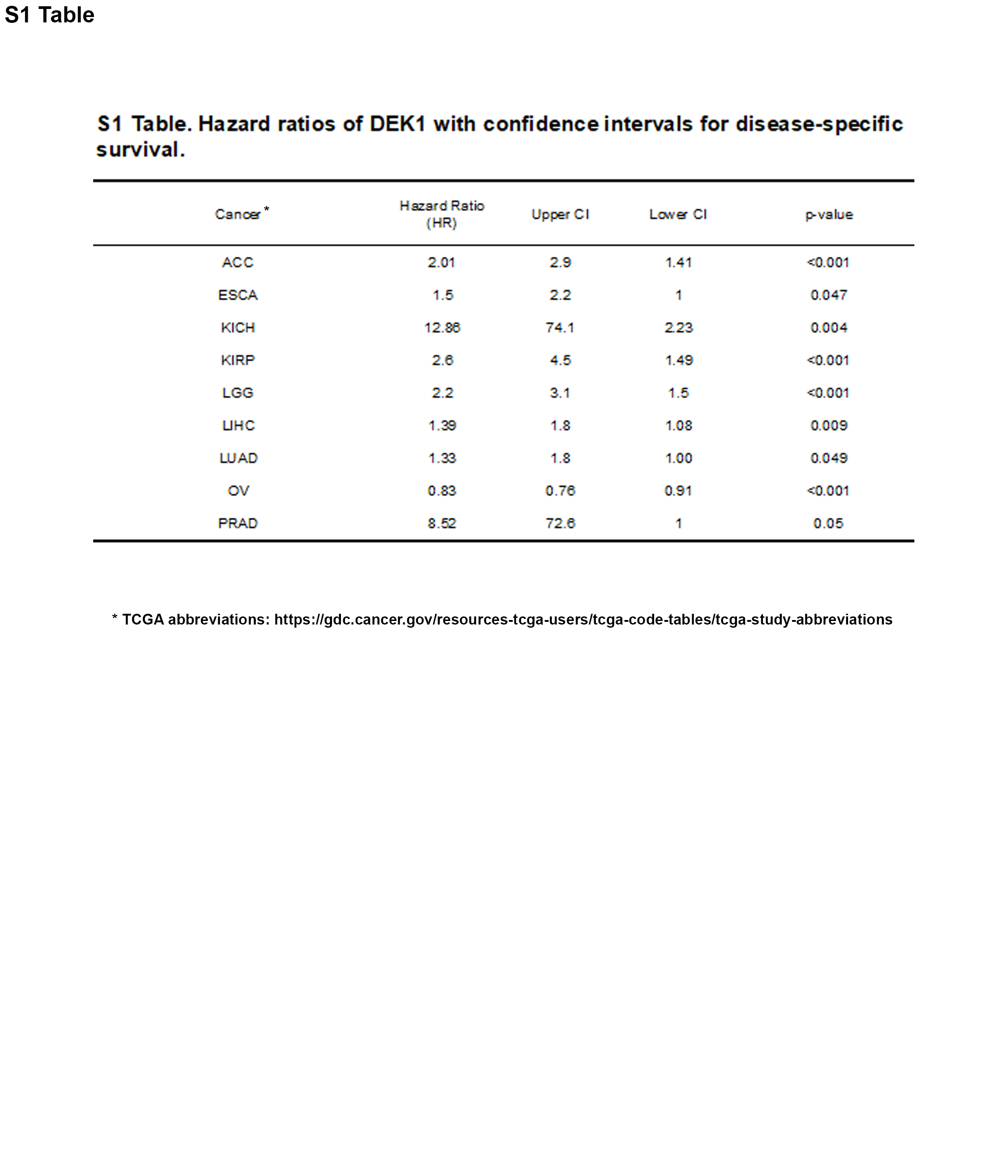

Supplement: S1 Table — (TIF) [file pone.0275476.s003.tif]

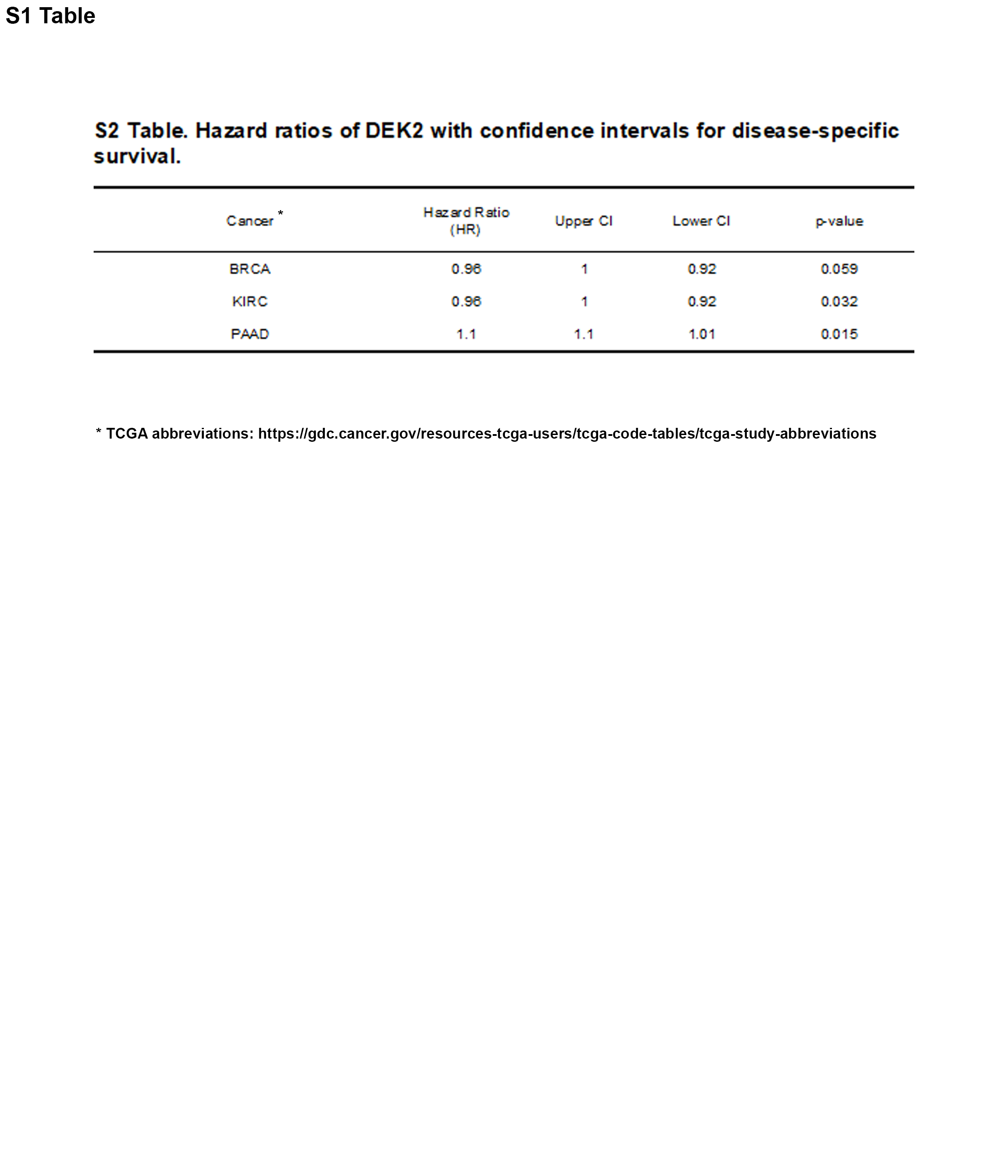

Supplement: S2 Table — (TIF) [file pone.0275476.s004.tif]
