# Supplementary material for: Doxorubicin induces prolonged DNA damage signal in cells overexpressing DEK isoform-2
Source: PLoS One. 2022 Oct 3;17(10):e0275476. doi: 10.1371/journal.pone.0275476 (PMC9529144; doi:10.1371/journal.pone.0275476)
Supplement: S1 Raw images — (PDF) [file pone.0275476.s005.pdf]

S1_raw_images

Figure 1A upper panel

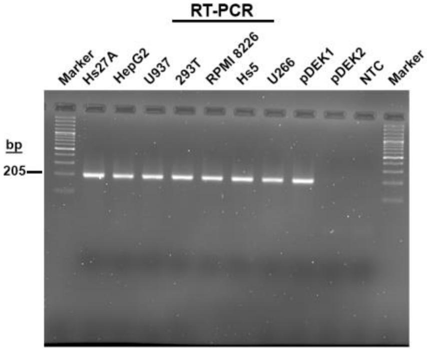

Figure 1A middle panel

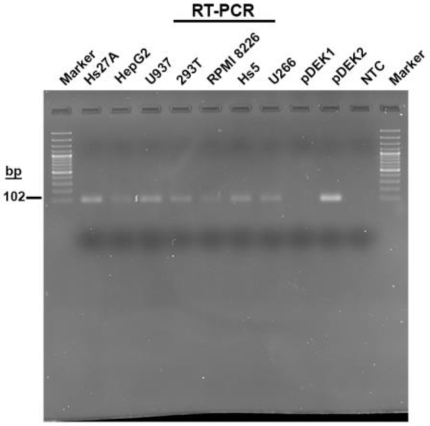

Figure 1A lower panel

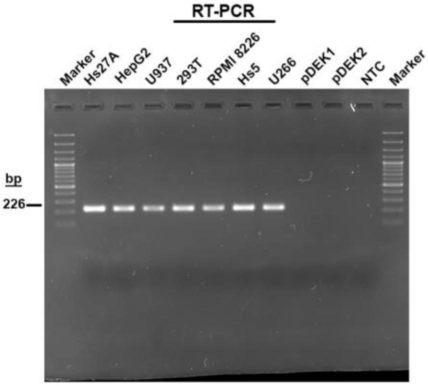

Figure 1D

Upper-left panel

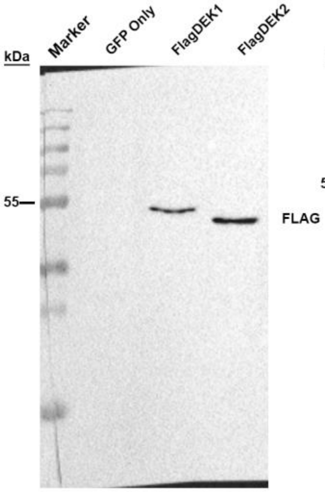

Upper-right panel

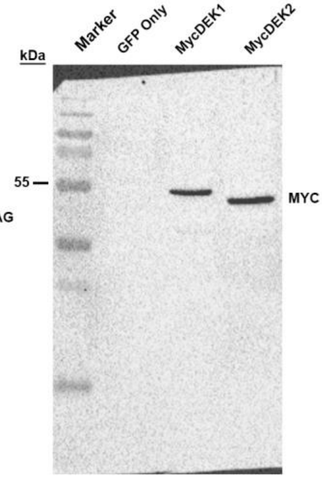

Lower-left panel

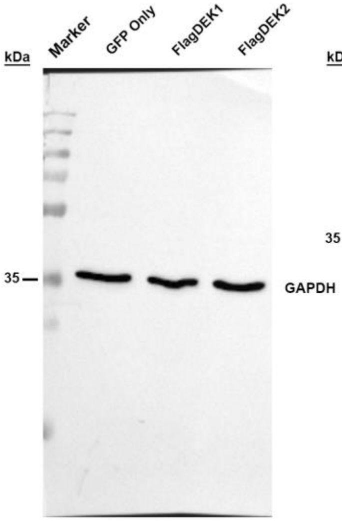

Lower-right panel

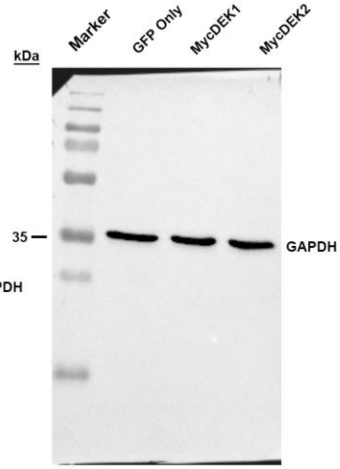

S1_raw_images

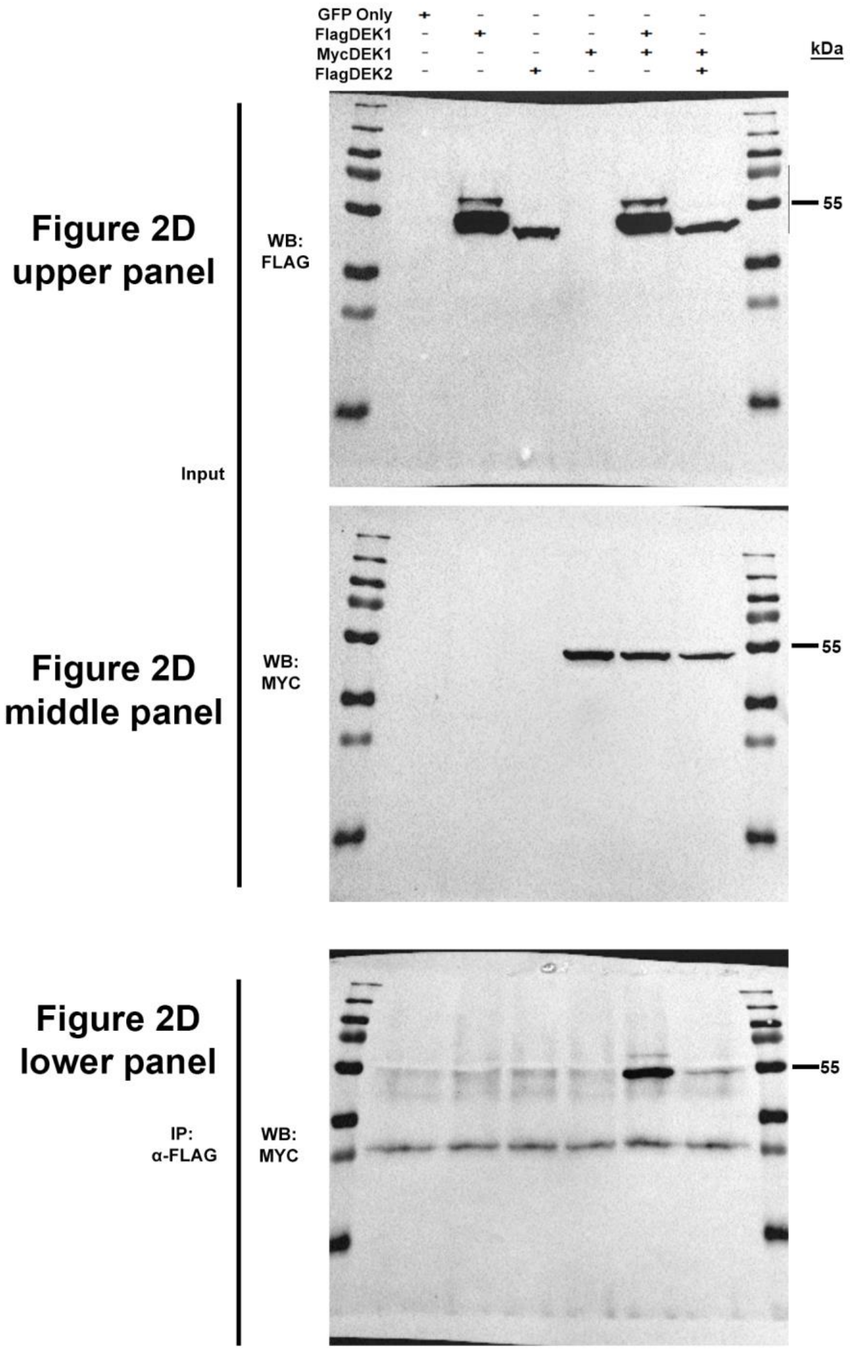

S1_raw_images

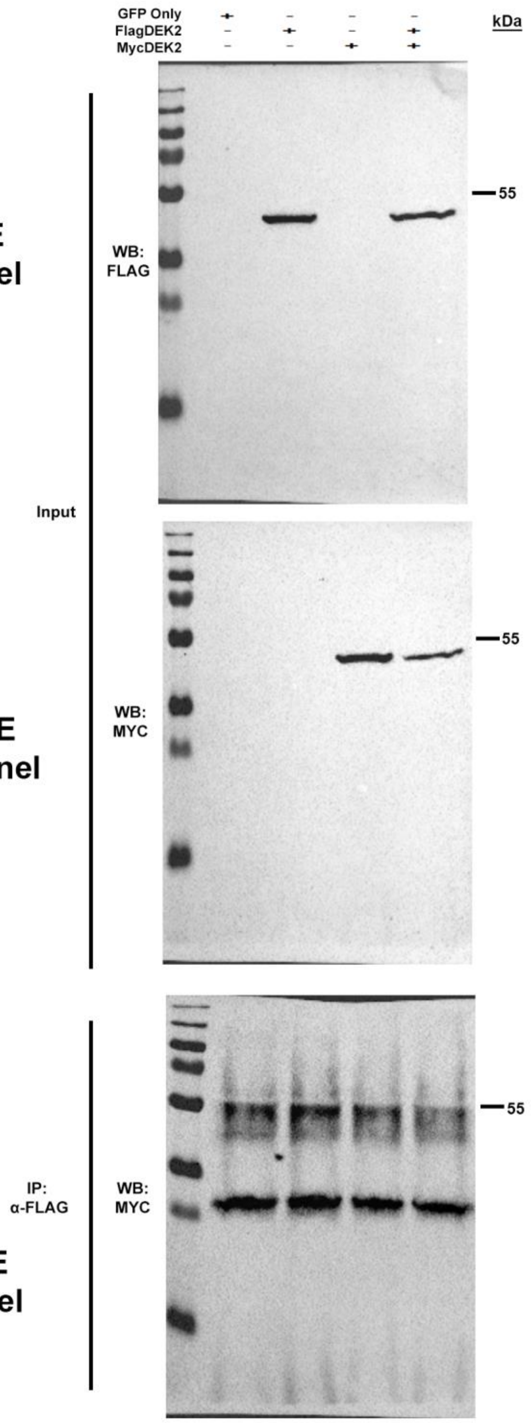

Figure 2E
upper panel

Figure 2E
middle panel

Figure 2E
lower panel

S1_raw_images

Figure 5E upper panel

Figure 5E lower panel

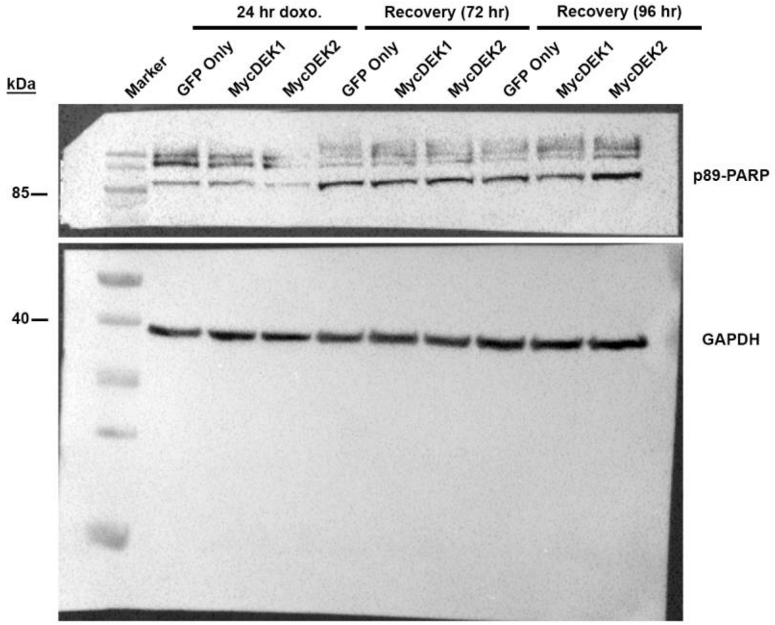

After transfer of the proteins, membrane was cut (as shown in upper and lower panel). Then membranes were probed with either p89-PARP antibody (upper panel) or with GAPDH antibody (lower panel).
