# Supplementary material for: Doxorubicin induces prolonged DNA damage signal in cells overexpressing DEK isoform-2
Source: PLoS One. 2022 Oct 3;17(10):e0275476. doi: 10.1371/journal.pone.0275476 (PMC9529144; doi:10.1371/journal.pone.0275476)
Supplement: Attachment — Submitted filename: Response to reviewers.docx [file pone.0275476.s006.docx]

September 2, 2022

Dear Editor,

We thank you and the reviewers for valuable comments about our manuscript (PONE-D-22-18362). We have answered the questions and we have revised the manuscript as suggested. Our response to the reviewer’s comments is shown in **blue**.

We hope this revision satisfactorily responds to all of the reviewers’ comments.

Yours sincerely,

Ayten Kandilci, Ph.D.

Gebze Technical University

Department of Molecular Biology and Genetics

Gebze, Kocaeli, Turkey.

**Journal Requirements:**

When submitting your revision, we need you to address these additional requirements.

**1.** Please ensure that your manuscript meets PLOS ONE's style requirements, including those for file naming. The PLOS ONE style templates can be found at

https://journals.plos.org/plosone/s/file?id=wjVg/PLOSOne_formatting_sample_main_body.pdf and

<https://journals.plos.org/plosone/s/file?id=ba62/PLOSOne_formatting_sample_title_authors_affiliations.pdf>

-**We have corrected the style in the revised manuscript as indicated in the PLOS ONE style templates.**

**2.** Thank you for stating the following in the Acknowledgments Section of your manuscript:

"This work was supported by the Scientific and Technological Research Council of Turkey (TÜBİTAK) (grant numbers 118Z765 and 216Z006). The authors thank Prof. Dr. Uygar Halis Tazebay for helpful discussions, Dr. Ömer Güllülü, Doğukan Metiner and Mehmet Soner Türküner for technical assistance."

We note that you have provided funding information that is not currently declared in your Funding Statement. However, funding information should not appear in the Acknowledgments section or other areas of your manuscript. We will only publish funding information present in the Funding Statement section of the online submission form. Please remove any funding-related text from the manuscript and let us know how you would like to update your Funding Statement. Currently, your Funding Statement reads as follows:

"Prof. Dr. Ayten Kandilci

Grant number: 118Z765

Funding: Scientific and Technological Research Council of Turkey (TÜBİTAK)

https://www.tubitak.gov.tr/

The funders had no role in study design, data collection and analysis, decision to publish, or preparation of the manuscript.

Mr. Emrah Özçelik

Grant number: 216Z006

Funding: Scientific and Technological Research Council of Turkey (TÜBİTAK)

https://www.tubitak.gov.tr/

The funders had no role in study design, data collection and analysis, decision to publish, or preparation of the manuscript."

-**We have removed funding-related text from Acknowledgments in the revised manuscript. Existing funding information in the “Funding Statement” section of the online submission form is enough and can be published as it is.**

**3.** We note that you have included the phrase “data not shown” in your manuscript. Unfortunately, this does not meet our data sharing requirements. PLOS does not permit references to inaccessible data. We require that authors provide all relevant data within the paper, Supporting Information files, or in an acceptable, public repository. Please add a citation to support this phrase or upload the data that corresponds with these findings to a stable repository (such as Figshare or Dryad) and provide and URLs, DOIs, or accession numbers that may be used to access these data. Or, if the data are not a core part of the research being presented in your study, we ask that you remove the phrase that refers to these data.

- **We removed the “data not shown” phrases in the revised manuscript and we provided the relevant data in the “Supporting Information Files” as “S1 Fig” and “S2 Fig”, and we cited the figures in the text (S1 Fig; lines 100 and S2 Fig line 367).**

4. PLOS ONE now requires that authors provide the original uncropped and unadjusted images underlying all blot or gel results reported in a submission’s figures or Supporting Information files. This policy and the journal’s other requirements for blot/gel reporting and figure preparation are described in detail at https://journals.plos.org/plosone/s/figures#loc-blot-and-gel-reporting-requirements and https://journals.plos.org/plosone/s/figures#loc-preparing-figures-from-image-files. When you submit your revised manuscript, please ensure that your figures adhere fully to these guidelines and provide the original underlying images for all blot or gel data reported in your submission. See the following link for instructions on providing the original image data: https://journals.plos.org/plosone/s/figures#loc-original-images-for-blots-and-gels.

In your cover letter, please note whether your blot/gel image data are in Supporting Information or posted at a public data repository, provide the repository URL if relevant, and provide specific details as to which raw blot/gel images, if any, are not available. Email us at plosone@plos.org if you have any questions.

**- As required by the journal, raw images were added to the “supporting information file” as “S1_raw_images”. We have noted that information in our “revision cover letter”.**

5. We note you have included a table to which you do not refer in the text of your manuscript. Please ensure that you refer to Table 2 in your text; if accepted, production will need this reference to link the reader to the Table.

**-We have corrected the table number in the revised manuscript and we referred as Table 2 in the text (lines 296 and 511).**

**Response to the Reviewers' comments:**

**Reviewer #1**: Özçelik et al. stably overexpressed DEK2 in the human bone marrow stromal cell line, and found that DEK2, unlike DEK1, is present in the nucleus and nucleolus, and that doxorubicin treatment causes persistent γH2AX signaling, which cannot functionally compensate for the loss of DEK1. Cells overexpressing DEK2 were found to be more sensitive to doxorubicin than DEK1 cells, and the expression of DEK1 and DEK2 in cell lines and primary tumors showed tissue specificity. In additon, only DEK2 is downregulated in a subset of breast cancers, suggesting that DEK2 may be differentially regulated from DEK1 in certain cancers. The authors concluded that the expression patterns and subcellular locations of the two DEK isoforms are different, suggesting that there is no overlap in function. This paper is well-written, however, some points are not clarified.

**1.** If DEK1 would be silenced, how does the DEK2 expression change?

**-This is an intriguing question. Since *DEK2*-mRNA is produced in the cells via alternative splicing of *DEK1*-mRNA, we think that silencing of *DEK1* using the sh-RNA approach that only targets the *DEK1*-mRNA would silence also the expression of *DEK2*-mRNA, given that shRNA can target the mRNA in the nucleus and in the cytoplasm (You et al. Cell Discovery (2021) 7:79** **https://doi.org/10.1038/s41421-021-00297-8). Therefore, we think that targeting specifically either only the *DEK1*-mRNA or both *DEK1* and *DEK2* mRNAs (as we did in the manuscript) would give rise to downregulation of endogenous *DEK2* expression. This hypothesis could be further supported by in silico findings that *DEK1* and *DEK2* are correlated in their expression based on TCGA cancer tissue and CCLE cell line datasets.**

2. In normal tissues, usually DEK1 expresses in the proliferative zone of the epithelium. The DEK2 expresses the same region? For example, how about colon (glandular) and esophagus (squamous cell) epitheliums?

**-As the reviewer stated, endogenous DEK1 protein is highly expressed in the proliferating epithelial cells therefore it could be important to evaluate endogenous DEK2 protein in these tissues. Unfortunately, there is not a DEK2-specific antibody, and commercially available anti-DEK antibodies do not discriminate between these two isoforms (because of that we have generated and ectopically expressed epitope-tagged DEK-isoforms in our study). In agreement with the comments of the reviewer, the generation of isoform-specific antibodies would be required to further evaluate/compare the endogenous DEK1 and DEK2 protein expression in the tissues or cells. It is important to note that, as we have shown in the manuscript, endogenous *DEK2* mRNA expression is very low compared to the endogenous *DEK1* mRNA in the cell lines that we have analyzed (Figs 1A and 1C; and DEK2:DEK1 ratio was about 1 to 250 in Fig 1C), which suggests that under steady-state conditions the level of endogenous DEK2 protein might also be below the detection limit of immunohistochemistry.**

**Based on the suggestions of the reviewer, in the revised manuscript, we emphasized why we used the overexpression system, and why we were not able to analyze endogenous DEK1 and DEK2 proteins (lines 61-69) as:**

*“Since there is not an antibody specific to the endogenous DEK2, currently it is not possible to analyze/discriminate endogenous DEK1 and DEK2 proteins using Western blot or immunofluorescent analyses. Therefore, here we elaborated the expression level of the two isoforms of DEK in selected cell lines and primary cancer samples using RT-qPCR and bioinformatics analysis of public datasets. We also compared the cellular location of ectopically expressed epitope-tagged DEK isoforms, and analyzed their effect on the proliferation, doxorubicin sensitivity, and DNA repair capacity in HS-27A cells, which relatively expresses higher endogenous DEK2.”*

3. It is interesting that only DEK2 is downregulated in a subset of breast cancers but not DEK1. Localization of two DEK proteins would be shown by double immunofluorescent staining in cell lines and/or human tissues.

**-We agree with the reviewer that data about the expression level of endogenous DEK-isoforms we obtained from “TCGA (RNA-seq)” platform is intriguing, and will form the basis of future projects. Unfortunately, it is not currently possible to show (or discriminate) the localization of two DEK-isoforms endogenously using double immunofluorescence staining since there are no isoform-specific antibodies. We added the former sentence to the revised manuscript (lines-61-63).**

**Reviewer #2:** This article explores the biology of a quite enigmatic variant of DEK: DEK2. The authors are investigating the similarities and differences between these 2 isoforms, and whether their expression levels could be of interest in cancer studies. Overall, this study is well conducted and well controlled. However, there are a few issues that the authors should consider revising.

**1.** The cloning section in the mat&met is confusing: was DEK2 cDNA obtained from cells or by site-directed mutagenesis using DEK1 cDNA? This point should be clarified.

**-We apologize for this confusing explanation. The *DEK2* cDNA was obtained from the *DEK1* cDNA using site-directed mutagenesis. We have corrected the relevant sentences in the revised manuscript (lines 72-80).**

**2.** Moreover, the bioinformatics analysis section in the mat&met is not sufficiently detailed and requires additional information such as which exact datasets have been used and their respective URL. Also, the identifier ENST00000397239.3 for DEK1 is incorrect and should be amended.

- **Thank you for this feedback. We have provided further detail on the materials and method in the bioinformatics analysis. We now give the URLs for XENA and depmap portal from which we obtained the isoform expression data for TCGA patient and CCLE cancer cell lines, respectively. Based on the most recent citing guidelines from we have added the following statement to the** **Acknowledgements section (line 551-552):**

*“The results shown here are in part based upon data generated by the TCGA Research Network:* *<https://www.cancer.gov/tcga>.”*

**We have further added the requested details on the transcript IDs and their respective Ensembl versions to clarify the differing ID numbers in the revised manuscript (lines 226-234):**

*“UCSC Xena uses the GENCODE v23 for transcript annotation hence the corresponding transcript IDs for the two DEK isoforms were matched with those from the newer GENCODE v39, based on their CCDS tags. Accordingly, the transcript ID for the full-length DEK1 for GENCODE v39, i.e.,, ENST00000652689.1, corresponded to ENST00000397239.7 in GENCODE v23. For DEK2, the transcript IDs from GENCODE v39 and v23 were the same (ENST00000244776.11). CCLE database also uses an archived version of Ensembl (http://grch37.ensembl.org/) for the transcript IDs of DEK1 and DEK2, i.e., ENST00000397239.3 and ENST00000244776.7, respectively.”*

**Moreover, we now have added the relevant references and explanations in text for ggpubr and ggbeeswarm packages of R that were used respectively for scatterplots and beeswarm plots used in the figures (lines 249-262).**

**3.** On line 258, Table 1 should be changed for Table 2.

**- We have corrected the table number (line 296).**

**4.** With figure 2B-D, the authors are willing to show the co-localisation between DEK2 and NPM1 in the nucleolus. However, from my point of view, the lack of NPM1 staining in figure 2C makes this panel irrelevant. The authors should consider adding NPM1 staining or removing this panel.

**-We thank for the suggestion. We agree, and we removed the corresponding (Fig 2C) panel. We uploaded the updated the figure 2.**

**5.** The authors are claiming that “DEK1-cells were resistant to doxorubicin-induced cell death” (lines 279-280). I do agree that the data showed that these cells are significantly less sensitive to doxorubicin, nevertheless they are still responsive to this treatment. Thus, I think this point should be amended.

**-We agree that the terms “less sensitive” rather than the “resistant” explains better our experimental results. Therefore, we have amended the corresponding sentence in the revised manuscript as shown below (lines 330-331):**

*“When we challenged the cells with doxorubicin (which induces DNA double-strand breaks; DSBs), we found that DEK1-cells were significantly less sensitive to doxorubicin treatment.”*

**-We also changed the conclusion sentence in the same paragraph (lines 334-337) as:**

*“These data suggest that the change in the expression balance between the two isoforms has different outcomes and a shift that increases only the DEK2 expression results in higher sensitivity to doxorubicin when compared to the DEK1 overexpression.”*

**6.** To the review question “2. Has the statistical analysis been performed appropriately and rigorously?” I answered “I don’t know” because I think a mistake was made in figure 4E. Indeed, all statistics indicate non-significant, but looking at the other panels and the text, I am convinced there are some significance there, isn’t it? Moreover, the authors are claiming on lines 313-314 that “the level of damage in DEK1-cells was higher than the DEK2-cells” but statistics are missing.

**- We added the statistics (Mann-Whitney test) to the figures 4D and 5B, and re-analyzed the statistics in figures 4E and 5C by using Two-way ANOVA Tukey’s multiple comparisons test. We uploaded these updated figures 4 and 5. We also edited the relevant sentences in the revised manuscript (lines 402-406) as:**

*“Similar experiments with DEK1 or DEK2 overexpressing cells (HS-27A-DEK1 and HS-27A-DEK2) indicated significantly more DNA damage after 24 hr of doxorubicin treatment in both group of cells compared to the control (HS-27A-GFP; GFP-only), and the level of damage in DEK1-cells was higher than the DEK2-cells, although statistically insignificant (Fig 5A and 5B). ”*

**7.** The authors only commented on the better recovery of DEK-1 cells compared to DEK2-cells. However, DEK1-cells present a significant reduction of gH2AX intensity compared to control-cells too. This point should be discussed.

**-We edited the corresponding paragraph in the revised manuscript (lines 406-409) as:**

*“Despite the higher DNA damage, DEK1-cells had a better repair capacity compared to both control and the DEK2-cells, although the difference was significant only between the DEK1 and DEK2 cells (Fig 5C).”*

**8.** Since p89-PARP expression presents only slight variations in figure 5E, it would be interesting to see a quantification.

**- We have revised the corresponding figure 5E and the figure legends (lines 440-442) and we added the quantification that we obtained by using Image-J software.**

**9.** Finally, DEK oncogene has already been studied in melanoma and hepatocellular carcinoma, for example. It would then be interesting to consider the potential involvement of DEK2 in the discussion.

**-** **We appreciate this suggestion. In the revised manuscript we now performed multivariable survival analysis for the 33 TCGA-PANCAN datasets, including melanoma and liver hepatocellular carcinoma, using a recently published online tool called SmulTCan (SmulTCan (bilkent.edu.tr)). SmulTCan can investigate the prognostic contribution of multiple genes/isoforms simultaneously in these 33 cancer types. When DEK1 and DEK2 mRNA expression levels were tested, there were only 9 cancers showing significant Hazard Ratios (HR) for DEK1 (S1_Table) and three (one approaching significance at p=0.059 for BRCA) for DEK2 (S2_Table). Interestingly in 8 out of 9 cancers DEK1 was significant which showed that higher expression of DEK1 indicated poor prognosis while patients in 2 out of 3 cancers where DEK2 was significant showed better prognosis. We have indicated the above findings in the results and discussion to suggest that DEK2 could be important and warrants further investigation in some cancers. We added Supplementary Tables 1 and 2 for the HR values (+/- confidence interval) and respective p-values that showed significance.**

**This information can be find in the revised manuscript (lines 464-471).**
